# A Proinflammatory Effect of the *β*-Glucan from *Pleurotus cornucopiae* Mushroom on Macrophage Action

**DOI:** 10.1155/2017/8402405

**Published:** 2017-05-15

**Authors:** Ken-ichiro Minato, Akihiro Ohara, Masashi Mizuno

**Affiliations:** ^1^Department of Applied Biological Chemistry, the Graduate School of Agriculture, Meijo University, Nagoya 468-8502, Japan; ^2^Graduate School of Agricultural Science, Kobe University, Kobe 657-8501, Japan

## Abstract

PCPS from *P. citrinopileatus* mushroom extract is a *β*-1,6-glucan possessing a proinflammatory effect on innate immune cells. The PCPS stimulated THP-1 macrophages to secrete significant levels of TNF. Moreover, the mRNA expressions of TNF and IL-1*β* were significantly enhanced by PCPS treatment. However, the PCPS did not induce to express both IL-12 and IL-10 mRNA in the macrophages. Next, the *P. cornucopiae* extract (containing mainly PCPS) treatment against mice showed significant increases in TNF and IL-1*β* mRNA expressions in the peritoneal macrophages of them. In this study, the expression levels of IFN*γ* mRNA in the spleen were almost the same between the extract- (PCPS-) treated group and control group. However, the expression of IL-4 mRNA showed a lower level in the extract-treated group than that in the control. Our results suggested that the PCPS could induce proinflammatory action in the immune response. In addition, the proinflammatory effect of the PCPS on THP-1 was enhanced by 5′-GMP-Na, while it was reduced by vitamin D_2_. These two compounds are majorly contained in the *P. citrinopileatus* mushroom. Therefore, these results suggested that the *P. citrinopileatus* mushroom might contain other immune regulative compounds, such as vitamin D_2_, as well as PCPS.

## 1. Introduction

Many studies have suggested immunomodulating effects of edible and medicinal mushroom. A lot of edible mushrooms have become attractive as “functional foods” and as source materials for immunomodulators, antitumor agents, antibiotics, and antihypertensive drugs [1–3]. Emerging studies suggest that specific compounds, such as polysaccharides, from those mushrooms have potent and unique properties as biological response modifiers (BRM). For example, the glucans, which are Lentinan, purified from the fruiting body of *Lentinus edodes*, as well as Krestin, isolated from a medicinal mushroom, *Trametes versicolor* [4] and D-fraction, a glucan from the edible mushroom *Grifola frondosa* [5], display an antitumor activity [6]. It is considered that these polysaccharides from edible and medicinal mushrooms could augment or complement a desired immune system to maintain a health condition in a host. Furthermore, it is proposed that differences in immunomodulating properties which are observed are due to differences in composition, size, and branching of the glucans through the interaction with multiple, different cellular receptors. The basidiomycete, *Pleurotus citrinopileatus*, called “oyster mushroom” is one of popular edible mushrooms in Japan. The production and the consumption of *P. citrinopileatus* is increasing rapidly [7], which raises great interest in its proposed immunomodulating properties and identification of the underlying molecular mechanisms. Recently, we have shown that *P. citrinopileatus* possesses *β*-1, 6-glucan with *β*-1, 3-glucoside side chains that can activate human DCs via triggering of dectin-1 [8].

Due to many immunological reports, it has been considered that a macrophage can play a key role in our immunomodulating action. Macrophages are dynamic and heterogeneous cells. They express various pattern recognition receptors (PRRs), such as Toll-like receptors and C-type lectins, and their interaction with a wide variety of different ligands leads to maturation, and/or modulation of the activation of macrophages, and the induction of specific adaptive immune responses [9–11]. PRR-activated macrophages will prime T cell responses. The macrophages initiate and regulate specific adaptive immune responses against various pathogens [12]. Moreover, it has been suggested that polarized macrophages are broadly classified into two types, M1 type and M2 type [13, 14]. Like Th1 lymphocytes, M1 is proinflammatory effectors and expresses mediators of inflammation such as IL-12, TNF, CC chemokines, and nitric oxide. In contrast, M2 macrophage plays an important role in immunoregulation system in which Th2 cytokines, such as IL-4 or IL-13 are involved. The monocytic THP-1 human myeloid leukemia cell line can be induced by phorbol 12-myristate 13-acetate (PMA) to differentiate into macrophage-like morphology [15]. Upon PMA treatment, the cells show an altered differentiation dependent on cell surface markers in a pattern similar to monocyte-derived macrophages [16]. In addition, they express a cytokine profile that resembles macrophages. We here set out to investigate the immune modulating properties of *P. citrinopileatus* glucan, PCPS, using macrophage-like cells derived from a THP-1 cell line, which are essential to regulate immune responses as well as DCs.

## 2. Materials and Methods

### 2.1. Reagent

*Escherichia coli* (0111:B4) LPS was purchased from Sigma-Aldrich (MO, USA). Human TNF mAb was purchased from R&D Systems (Abingdon, U.K.). qPCR was performed by using SYBR^®^ Premix EX Taq™ II (Takara, Japan). 5′-GMP-Na and ergocalciferol (as vitamin D_2_) were purchased from Sigma-Aldrich (MO, USA).

### 2.2. Preparation of the Mushroom Extracts

The 450 kD polysaccharide fraction of *P. citrinopileatus* was prepared from a hot water extract as described previously [7]. Shortly, a hot water extract (a crude glucan fraction) from the mushroom fruiting body was separated into six fractions by DEAE-Sepharose chromatography. The active fraction was further purified by using a Sephacryl S-400 column, resulting in the active 450 kDa polysaccharide named PCPS, which was dissolved in distilled H_2_O. The amount of PCPS in the hot water extract was 735.4 ± 43.5 *μ*g/mg (as glucose, measured by anthrone-sulfuric acid method). To determine whether PCPS was endotoxin free, the polysaccharide was pretreated with polymyxin B (10 *μ*g/ml, Sigma-Aldrich, MO, USA) for 2 hours at 37°C prior to the priming.

### 2.3. Preparation of Macrophage Cell Line

The monocyte cell-line, THP-1 (Riken BRC Cell Bank, Tsukuba, Japan) was cultured to confluence in RPMI1640 medium containing 10% fetal bovine serum (FBS) and 1% glutamate/penicillin/streptomycin (G/P/S). THP-1 cells were differentiated to macrophages as follows. Their macrophage-like states were obtained by treating each monocyte for 48 hrs with 160 nM of phorbol 12-myristate 13-acetate (PMA; Sigma) in 24-well cell culture plates (Nunc) with cell suspension (1 × 10^6^ cells). Then, differentiated and adherent cells were washed twice with RPMI 1640 medium containing 10% FBS and 1% G/P/S. And, to obtain the resting state of macrophage, they were rested for another 24 hr in the culture medium. After incubation, the cultures were washed to remove the nonadherent cells, and then the tested sample in fresh medium was added to the plates and cultured for 24 hr at 37°C and 5% CO_2_. Then, the supernatant of cultures was collected and kept at −80°C until a measurement of the cytokine production.

### 2.4. Determination of TNF Level Secreted by the Differentiated THP-1 Macrophage

The production of TNF by the differentiated THP-1 macrophages was determined by ELISA analysis of the cell-free supernatants after incubation of the THP-1 with different stimuli for 24 hours. TNF level was determined by using anti-TNF antibodies as previously described [17]. Briefly, a NUNC maxisorb 96-well plate was coated with coating Ab of each cytokine overnight at 4°C. After blocking for 30 min at 37°C, the supernatant of the culture and the detection Ab was added (2 hours, on an orbital shaker at 500 rpm, RT). Simultaneously, cytokine concentration standards were tested. The plate was washed 4 times with PBS and incubated for 30 min with Streptavidin HRP. The reaction was developed by Tetramethylbenzidine (TMB) substrate, and optical density was measured by a spectrophotometer (450 nm).

### 2.5. The *P. citrinopileatus* Extract Treatment for the Mice

All animal experiments were performed in accordance with the guidelines of the Animal Care and Use Committee of Meijo University and were approved by the Committee. BALB/c mice (6-week-old, female) were purchased from Japan SLC Inc. (Shizuoka, Japan). The animals were housed in controlled conditions (20–25°C, 50–55% humidity, and a 12 h light/dark cycle) with free access to water and a standard rodent chow (AIN-76A, Research Diet Inc.). After acclimation for 7 days, the animals were randomly divided into 2 groups (*n* = 5 for each group): normal control (control), *P. citrinopileatus* extract (containing mainly PCPS) group (680 *μ*g/kg b.w. as 500 *μ*g/kg based on PCPS). The experimental extracts dissolved in distilled water were orally administered every day for two weeks. Normal control mice were administered an equal volume of distilled water. Body weights were monitored every two days during the period. At necropsy, peritoneal cells and spleen were collected under inhalation anesthesia. Peritoneal cells were collected by repeated intraperitoneally wash of RPMI medium. Collected cells were washed with RPMI 1640 and then plated in 24-well culture plates at a density of 1 × 10^6^ cells per well. Cultures were incubated for adhesion to plates for 2 hrs at 37°C and 5% CO_2_. After incubation, the cultures were washed to remove the nonadherent cells. Then fresh medium was added to the plates and cultured for 2 hrs at 37°C and 5% CO_2_. After incubation, to measure cytokine mRNA level expressed in the macrophages, the cells were collected and then stored in RNAlater^®^ (Qiagen) at −80°C until a measurement of cytokine mRNA expression. Spleen in each mouse was collected, immediately followed by addition of RNAlater. And then they were stored at −80°C until a measurement of cytokine mRNA expression.

### 2.6. Quantitative Real-Time PCR

The total RNA extractions from cultured frozen mouse spleen were performed by using RNAeasy^®^ reagent (Qiagen) according to the manufacturer's protocol. And then cDNAs were synthesized from the total RNAs by reverse transcription by using PrimeScript^®^ RT Master Mix (Takara, Japan) priming with random hexamer and oligo (dT), according to the manufacturer's instructions. Oligonucleotide primers (Table 1) were designed as described previously [7, 8] and purchased as commercially available products (STAR Oligo, Rikaken, Japan). Real-time PCR were performed with SYBR Green method as described previously using StepOne Plus™ Real-Time PCR System (ThermoFisher Scientific) [18]. Relative changes in mRNA expression between samples were determined using the comparative Ct method (ΔΔCt).

### 2.7. Statistical Analysis

Results are expressed as the mean ± S.E.M. Statistical analyses were performed using SPSS 24 software (IBM, USA). A one-way ANOVA was performed followed by the Dunnett's *t* test, or Student's *t* test was performed. The level of significance was stated in the figure legends.

## 3. Results

### 3.1. The PCPS Shows the Potential to Induce Proinflammatory Cytokine Secretion in the Differentiated THP-1

**Figure 1:**
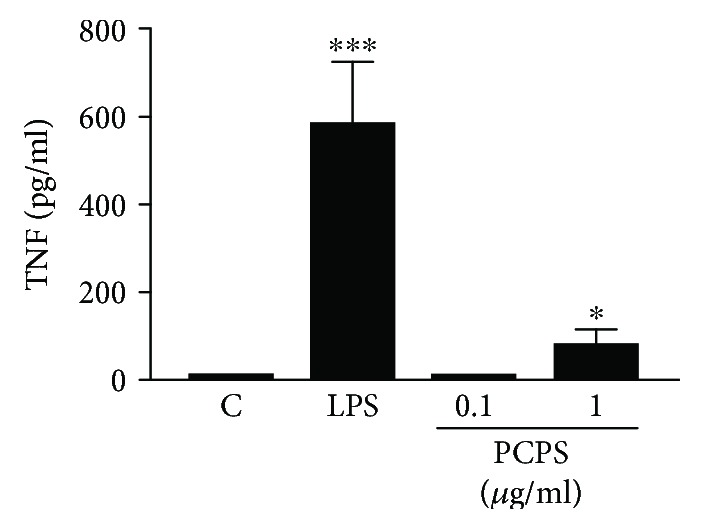
*P. citrinopileatus* polysaccharide PCPS induces secretion of proinflammatory cytokine, TNF, in THP-1 macrophages. Human monocyte cell line THP-1 was differentiated into macrophages by PMA (160 nM) for 48 hrs., and then they were stimulated with the different concentrations of the 450 kD polysaccharide (PCPS) purified from a hot water extract of *P. citrinopileatus* fruiting bodies, or LPS (10 ng/ml, as a positive control). After 24 hours, the secretion of TNF in the cell supernatant was determined by ELISA. The data shown are the average of 3 independent experiments. The depicted error bars represent the SEM of these 3 experiments. For statistical analysis, a one-way ANOVA followed by the Dunnett's *t* test was used; ^∗^*p* < 0.05 and ^∗∗∗^*p* < 0.001 when compared to the RPMI medium control.

**Figure 2:**
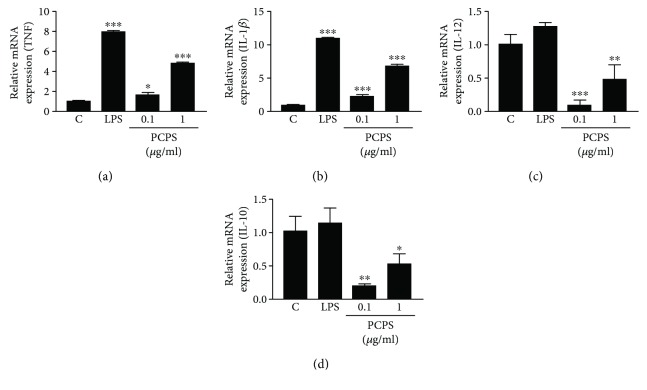
The *P. citrinopileatus* polysaccharide PCPS induces some proinflammatory cytokine expressions in THP-1. Human monocyte cell line THP-1 was differentiated into macrophages by PMA (160 nM) for 48 hrs., and then they were stimulated with the different concentrations of the 450 kD polysaccharide (PCPS) purified from a hot water extract of *P. citrinopileatus* fruiting bodies, or LPS (10 ng/ml, as a positive control). After 2 hours, the mRNA expression levels of TNF (a), IL-1*β* (b), IL-12 (c), and IL-10 (d) in the THP-1 were determined by using quantitative real-time PCR. Expression was determined relative to glyceraldehyde 3-phosphate dehydrogenase (GAPDH) and then each expression level was compared to the level of the RPMI medium control. The experiments shown are an average of 3 experiments. The depicted error bars represent the SEM of these 3 experiments. For statistical analysis, a one-way ANOVA followed by the Dunnett's *t* test was used; ^∗^*p* < 0.05, ^∗∗^*p* < 0.01, and ^∗∗∗^*p* < 0.001 when compared to the RPMI medium control.

A 450 kD *P. citrinopileatus* polysaccharide (PCPS) was obtained from a hot water extract of the mushroom's fruiting bodies, followed by ethanol precipitation and purification steps as described previously [7]. It has been shown that the PCPS contains *β*-1, 6-glucopyranoside residues as (part of) the main chain by the observation of the ^13^C NMR signal and suggested that *β*-1, 3-branched glucopyranoside residues are attached to the main structure of PCPS [8]. To determine whether PCPS could activate macrophages, the differentiated macrophage-like cells, THP-1, by PMA-treatment were incubated with this glucan. PCPS stimulated the macrophages to secrete significant levels of the proinflammatory mediators TNF (Figures 1 and 2(a)). In addition, cytokine mRNA levels were determined in the differentiated THP-1 stimulated with PCPS or LPS (as a positive control) for 2 hours (Figure 2). The mRNA expression levels of TNF (Figure 2(a)) and IL-1*β* (Figure 2(b)) were significantly enhanced in the THP-1 stimulated with PCPS. However, PCPS stimulation did not induce secretions of both IL-12 (Figure 2(c)) and the anti-inflammatory cytokine, IL-10, in the matured macrophages (Figure 2(d)). To exclude the contamination of endotoxin in the PCPS polymyxin B (20 mg/ml), an antibiotic that binds lipid A in LPS was included in the assays. Addition of polymyxin B in the assay did not affect the cytokine secretion stimulated by PCPS, although it could completely inhibit the LPS-induced production of TNF in the macrophages (data not shown). These results show that PCPS can activate matured macrophages to secrete proinflammatory cytokines, TNF, and IL-1*β*, in the absence of an endotoxin, but not IL-12.

### 3.2. Effects of the Mushroom Extract on mRNA Expression of Macrophage Cytokine in Mice

**Figure 3:**
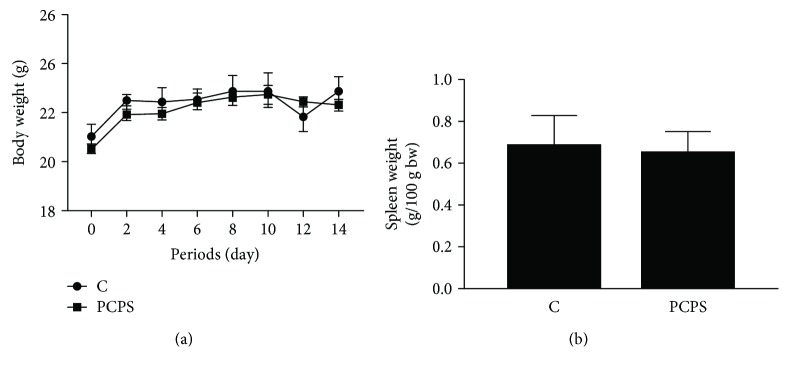
Effects of the *P. citrinopileatus* extract on body weights and relative spleen weights. The depicted error bars represent the SEM (*n* = 5/group). After acclimation for 7 days, the animals were randomly divided into 2 groups (*n* = 5 for each group): normal control (C), *P. citrinopileatus* extract group (PCPS). The experimental extracts dissolved in distilled water were orally administered every day for two weeks. Normal control mice were administered an equal volume of distilled water. Body weights were monitored every two days during the period. Spleen weights were calculated as a proportion of body weight (g/100 g bw) at day 14.

**Figure 4:**
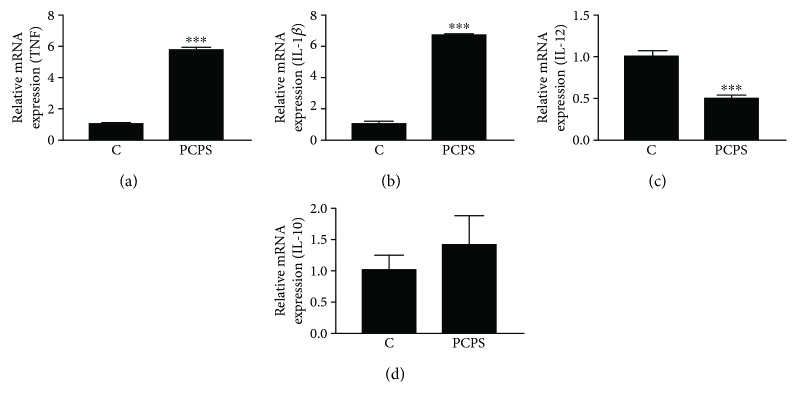
Effects of the *P. citrinopileatus* extract on cytokine expression in the mice peritoneal cells. After acclimation for 7 days, the animals were randomly divided into 2 groups (*n* = 5 for each group): normal control (C), *P. citrinopileatus* extract group (PCPS). The experimental extracts dissolved in distilled water were orally administered every day for two weeks. Normal control mice were administered an equal volume of distilled water. Peritoneal cells in the treated mice were collected after thioglycollate injection. After 2 hours incubation, the mRNA expression levels of TNF (a), IL-1*β* (b), IL-12 (c), and IL-10 (d) in the adherent cells from the peritoneal cells were determined by using quantitative real-time PCR. Expression was determined relative to glyceraldehyde 3-phosphate dehydrogenase (GAPDH), and then each expression level was compared to the level of the normal control group. The depicted error bars represent the SEM, and Student's *t* test was used for the statistical analysis; ^∗∗∗^*p* < 0.001 when compared to the normal control.

The changes in body weights of the mice are presented in Figure 3. No significant change in the body weights was observed between both groups throughout the two-week feeding period. Peritoneal cells in the treated mice were collected after thioglycollate injection. The *P. cornucopiae* extract- (PCPS-) treated group showed significant increase in TNF and IL-1*β* mRNA levels in the attached cells compared to the normal control group (Figures 4(a) and 4(b)). However, IL-12 mRNA of the extract- (PCPS-) treated mice expressed at lower than that of control mice peritoneal cells (Figure 4(c)). Meanwhile, the extract treatment did not show a significant increase in the expression of IL-10 mRNA in the attached peritoneal cells (Figure 4(d)). These results suggested that the *P. cornucopiae* extract (PCPS) treatment predominantly showed the effect on proinflammatory action of immunocompetent cells in the peritoneal.

### 3.3. Effects of the Mushroom Extract on Cytokine mRNA Expressions in the Spleen of the Treated Mice

**Figure 5:**
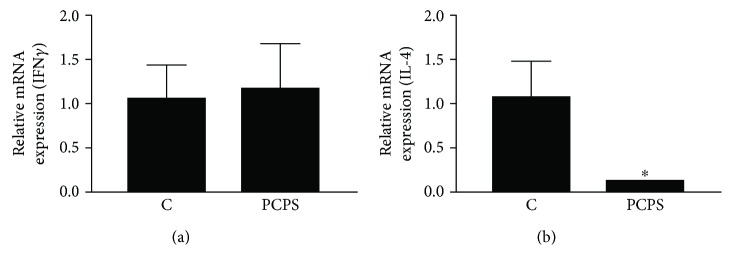
Influences of the *P. citrinopileatus* extract on cytokine expression in the mice spleen cells. After acclimation for 7 days, the animals were randomly divided into 2 groups (*n* = 5 for each group): normal control (C), *P. citrinopileatus* extract group (PCPS). The experimental extracts dissolved in distilled water were orally administered every day for two weeks. Normal control mice were administered an equal volume of distilled water. The mRNA expression levels of IFN*γ* (Figure 5(a)) and IL-4 (Figure 5(b)) in the mice spleen cells were determined by using quantitative real-time PCR. Expression was determined relative to glyceraldehyde 3-phosphate dehydrogenase (GAPDH), and then each expression level was compared to the level of the normal control group. The depicted error bars represent the SEM, and Student's *t* test was used for the statistical analysis; ^∗^*p* < 0.05 when compared to the normal control.

The change in spleen weights (calculated as a proportion of body weight) is also presented in Figure 3. No significant change in the spleen weights was observed between both groups during the feeding period. As shown in Figure 5(a), expression levels of IFN*γ* mRNA in the spleen cells were almost the same between the *P. cornucopiae* extract- (PCPS-) treated mice group and control group. However, the expression of IL-4, anti-inflammatory cytokine, mRNA showed a lower level in the spleen of the extract-treated group compared to that of the normal control group (Figure 5(b)). Moreover, it was shown that the *P. cornucopiae* extract treatment (PCPS group) induced an increase in CD3^+^CD4^+^ cell population in the spleen by using FACS analysis (data not shown). These results suggested that the PCPS might inhibit anti-inflammatory action of the spleen lymphocyte.

### 3.4. Combination Effect of Guanyl Acid/Vitamin D_2_ with PCPS on Proinflammatory Cytokine Secretion from the Stimulated Macrophages

**Figure 6:**
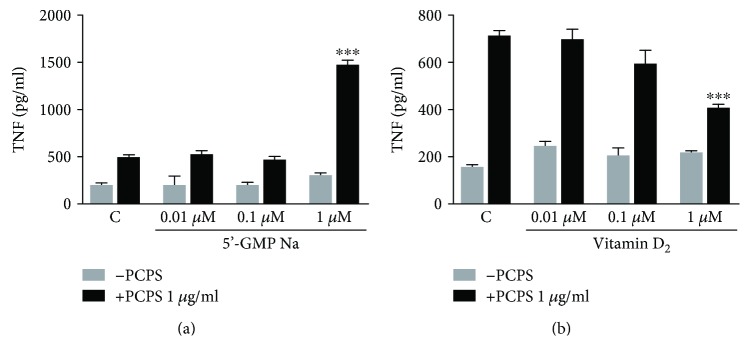
Combination effect of guanyl acid/vitamin D_2_ with PCPS on proinflammatory cytokines secretion from the stimulated macrophages THP-1. Human monocyte cell line THP-1 was differentiated into macrophages by PMA (160 nM) for 48 hrs., and then they were stimulated with 1 *μ*g/ml of the 450 kD polysaccharide (PCPS) purified from a hot water extract of *P. citrinopileatus* fruiting bodies plus the different concentrations of 5′-GMP-Na (Figure 6(a)) or ergocalciferol as vitamin D_2_ (Figure 6(b)). After 24 hours, the secretion of TNF in the cell supernatant was determined by ELISA. The data shown are the average of 3 independent experiments. The depicted error bars represent the SEM of these 3 experiments. For statistical analysis, a one-way ANOVA followed by the Dunnett's *t* test was used; ^∗∗∗^*p* < 0.001 when compared to the RPMI medium control.

The proinflammatory effect of the PCPS on THP-1 activity was enhanced by 5′-GMP-Na (Figure 6(a)), while it was reduced by vitamin D_2_ (Figure 6(b)). These results suggested that 5′-GMP-Na could upregulate PCPS-induced proinflammatory action; however, ergocalciferol would downregulate that.

## 4. Discussion

Various natural products have been used to treat and/or prevent many kinds of diseases, and the treatment of them has a long therapeutic history. Especially, natural *β*-glucans isolated from yeast, grain, and mushrooms are well-established biological response modifiers [19]. The glucans are highly conserved carbohydrates and possess a group of chemically heterogeneous polysaccharides polymerizing with various numbers of molecules of glucose bound together with several types and degrees of branching. The glucan research began with showing its ability to stimulate the phagocytic system, enhance general defense mechanisms, and promote resistance to tumours. The effects of many glucans, which are able to activate macrophages, have been already reported, and their effects play an important role in the immune response [20–22]. *β*-glucans, such as lentinan and D-fraction, have shown to exert beneficial therapeutic effects against various diseases. Currently, several additional important effects of the glucans have demonstrated, including reduction of stress [23], hypoglycemic effects, reduction of cholesterol level [24], and improvements of ulcerative colitis [25]. Moreover, the glucans were found to significantly stimulate defense reactions against infections and cancer [26]. Furthermore, *β*-glucan administration could prevent/improve symptoms of allergic rhinitis and upper respiratory tract infections [27]. We previously isolated an immunomodulating 450 kDa polysaccharide from the oyster mushroom *P. citrinopileatus* (PCPS) which was composed of glucose [7]. Our structural analysis of *P. citrinopileatus* PCPS suggested that PCPS most likely is a *β*-1, 3-branched *β*-1, 6-glucan [8]. Other reports demonstrate immunomodulating *β*-1, 6-branched *β*-1, 3-D-glucan polysaccharides in Pleurotus mushrooms [28], suggesting that Oyster mushrooms contain different types of immunomodulating *β*-glucans.

We have already reported that PCPS can induce maturation of human immature DCs and the secretion and/or expression of many proinflammatory mediators, as well as the secretion of the anti-inflammatory cytokine IL-10 [8]. Our findings suggested that PCPS can induce activation of DCs, but may simultaneously suppress (over) production of proinflammatory mediators which may contribute to a balanced immune response in their hosts. Furthermore, we here showed that PCPS treatment could stimulate proinflammatory action of macrophage in vivo as well as in vitro (Figures 1, 2, and 4). Because it is generally considered that maintaining a balance of M1 and M2 macrophages could prevent from a development of an immune-deficient disease, such as inflammatory, allergy, and cancer, it is certainly expected that the immunomodulating polysaccharide could regulate this macrophage balance. In this study, we showed that PCPS possessed proinflammatory effect on macrophage action. However, our recent study focused on the influence of PCPS on action of innate immune system. Indeed, macrophages, which are widely distributed in a variety of organs [29], play important roles in regulating responses among each cell in an immune system [30]. Therefore, it remains unclear what kind of influence the PCPS treatment shows on action of lymphocytes in acquired immune system.

PRR-activated innate immune cells such as macrophages and DCs will prime T cell responses. Regarding macrophage, mirroring the Th1/Th2 polarization of T cells, polarized macrophages are often referred to as inflammatory (M1 or classically activated) and regulatory (M2 or alternatively activated) macrophages [13, 31]. Inflammatory macrophages are involved in antimicrobial activities. In contrast, it has also been reported that regulatory macrophages promote tissue repair and contribute to metabolic homeostasis, and they are involved in immunity against parasitic helminth infections, through an acquired immune system [29, 32]. Moreover, regarding amelioration of allergic diseases, such as allergic rhinitis, asthma, and chronic atopic disorders, it is important to control the Th2 immune response [33, 34]. Therefore, it is important to examine whether immunomodulating agents such as *β*-glucan have influence on actions of lymphocytes such as T cell populations in acquired immune system, as well as that on macrophage action in innate immune system. In this study, it was shown that the *P. cornucopiae* extract treatment (PCPS group) induced an increase in CD3^+^CD4^+^ cell (as helper T lymphocytes) population in the spleen. However, the population of CD3^+^CD8^+^ (as killer T lymphocytes) has not been affected by PCPS treatment in the spleen of the mice (data not shown). The PCPS treatment inhibited an expression of IL-4, an anti-inflammatory cytokine, mRNA in the lymphocytes of the mice spleen in this study. Meanwhile, interestingly, the glucan treatment did not show any influence on an expression of IFN*γ* mRNA in the spleen, which is a proinflammatory cytokine (Figure 5). In the immune system, the cytokines IL-12, TNF, and IL-1*β* [35] are known as key effectors linked to host protection and secreted by both innate and acquired immune cells. Among them, IL-12 is critical for the induction of IFN*γ* in a variety of immune cells as well as the activation of CD4^+^ T cells, leading to the protective Th1 response [36]. The proinflammatory effect of PCPS on IL-12 secretion was significantly weak in the mice peritoneal macrophages stimulated with PCPS, as well as in the THP-1 (Figure 4). Therefore, the secretion of IFN*γ* might not enhance in the spleen cells of treated mice.

The mushroom contains many kinds of bioactive substances such as vitamin D_2_, nucleic acids, and amino acid as well as *β*-glucan. Some reports have shown that the effect of glucan was synergistically increased by combination dose of other substances. Therefore, in this study, we attended to assess the combination effect of PCPS and some substances contained in *P. citrinopileatus* mushroom. The effect of the PCPS on proinflammatory action of macrophage was enhanced by 5′-GMP-Na, while it was reduced by vitamin D_2_ treatment (Figure 6). It has been known that both 5′-GMP-Na and vitamin D_2_, ergocalciferol, are major substances in the mushrooms. These compounds, in our study, might affect the proinflammatory effect of single PCPS in systemic immune response. In this study, combination dose of PCPS with 5′-GMP-Na/vitamin D_2_ showed to affect proinflammatory action of macrophages such as TNF secretion. Therefore, our results suggested that the *P. citrinopileatus β*-glucan could regulate macrophage activities, and the mushroom might contain other immune regulative compounds, such as vitamin D_2_, for prevention from excess proinflammatory activities.

By the way, we have showed that the signalling pathways via not only dectin-1 but also TLRs play major roles in the PCPS-mediated immune response, suggesting PCPS has the capacity to activate human DCs via multiple pathways [8]. Several studies have demonstrated that the cross-talk between dectin-1 and TLRs is required for activation of NF-κB and production of inflammatory cytokines in monocytes, DCs, and macrophages [37, 38], suggesting that collaboration between dectin-1 and the TLRs might play an important role in inflammatory responses to fungus polysaccharide. Interestingly, PCPS could also induce secretion of the anti-inflammatory cytokine, IL-10, in DCs [8]. And, the effect of PCPS on innate immune cells and on acquired immune cells, in this study, was not consistent with each other. Detailed studies of single treatment of the *P. citrinopileatus* polysaccharide PCPS will be necessary to completely understand how this polysaccharide modulates the immune response.

## 5. Conclusion

The *P. citrinopileatus* polysaccharide PCPS has been identified as a *β*-glucan which activates macrophage cells by upregulation of the secretion or expression of many proinflammatory mediators. It has been shown that the PCPS from *P. citrinopileatus* extract contains *β*-1, 6-glucopyranoside residues as (part of) the main chain with *β*-1, 3-branched glucopyranoside residues. PCPS from *P. citrinopileatus* mushroom extract is a *β*-1, 6-glucan possessing a proinflammatory effect on innate immune cells. The PCPS stimulated THP-1 macrophages to secrete significant levels of TNF. Moreover, the mRNA expressions of TNF and IL-1*β* were significantly enhanced by PCPS treatment. However, the PCPS did not induce to express both IL-12 and IL-10 mRNA in the macrophages. Next, the *P. cornucopiae* extract (containing mainly PCPS) treatment against mice showed significant increases in TNF and IL-1*β* mRNA expressions in the peritoneal macrophages of them. These results suggested that the PCPS could induce proinflammatory action in an innate immune response. Meanwhile, the expression of IL-4 mRNA showed a lower level in the extract-treated group than that in the control. However, the expression levels of IFN*γ* mRNA in the spleen cells were almost the same between the extract- (PCPS-) treated group and control group. Further studies of the effect of *P. citrinopileatus* polysaccharide PCPS on those cytokine expressions will be necessary to completely understand how this polysaccharide modulates the immune response between IL-4 and IFN*γ*. In addition, the proinflammatory effect of the PCPS on THP-1 was enhanced by 5′-GMP-Na, while it was reduced by vitamin D_2_. These two compounds are majorly contained in the *P. citrinopileatus* mushroom. Therefore, our results suggested that the *P. citrinopileatus* mushroom could regulate macrophage activities by its *β*-glucan and might contain other immune regulative compounds, such as vitamin D_2_, as well as PCPS.

## Figures and Tables

**Table 1:** Oligonucleotide primers designed for human (h) or mouse (m) genes in quantitative real-time PCR.

Gene	Forward (5′- 3′)	Reverse (5′- 3′)
*hTNF*	gaggccaagccctggtatg	cgggccgattgatctcagc
*hIL-1b*	atgatggcttattacagtggcaa	gtcggagattcgtagctgga
*hIL-10*	gactttaagggttacctgggttg	tcacatgcgccttgatgtctg
*hIL-12p40*	gcggagctgctacactctc	ccatgacctcaatgggcagac
*hGAPDH*	acgaccccttcattgacc	agacaccgatagactccacg
*mTNF*	caggcggtgcctatgtctc	cgatcaccccgaagttcagtag
*mIL-1b*	gaaatgccaccttttgacagtg	tggatgctctcatcaggacag
*mIL-4*	ccccagctagttgtcatcctg	caagtgatttttgtcgcatccg
*mIL-10*	gtgaaaataagagcaaggcagtg	attcatggccttgtagacacc
*mIFNg*	agcggctgactgaactcagattgtag	gtcacagttttcagctgtataggg
*mGAPDH*	tcaacagcaactcccactcttcca	acctgttgctgtagccgtattca
